# Does the Use of the Yeast Probiotic *Saccharomyces cerevisiae* Actisaf Sc 47 Reduce the Environmental Impacts of Beef Cattle? A Study Based on Life Cycle Assessment

**DOI:** 10.3390/ani14213107

**Published:** 2024-10-28

**Authors:** Nizar Salah, Héloïse Legendre, Erika Paiva, Julie Duclos, Maxime Briche, Florine Colbalchini, Armelle Gac, Thomas Kerihuel, Céline Garat Boute

**Affiliations:** 1 Phileo by Lesaffre, 59520 Marquette-lez-Lille, Francee.paiva@phileo.lesaffre.com (E.P.); j.duclos@phileo.lesaffre.com (J.D.); m.briche@phileo.lesaffre.com (M.B.); c.garatboute@phileo.lesaffre.com (C.G.B.); 2 EVEA, 11 Rue Voltaire, 44000 Nantes, France; f.colbalchini@evea-conseil.com (F.C.); a.gac@evea-conseil.com (A.G.); t.kerihuel@evea-conseil.com (T.K.)

**Keywords:** yeast probiotic, Actisaf^®^ Sc 47, life cycle assessment, beef cattle, fattening period

## Abstract

Increasing animal protein, especially from beef, is necessary to satisfy the requirements of the growing global population. However, this is becoming increasingly challenging due to stakeholders prioritizing sustainable systems with low environmental impacts. To answer these challenges, the use of feed additives such as yeast probiotics seems to be an effective route for improvements in feed efficiency and growth performance. The current study was performed to quantify the environmental impacts of the production and use of a yeast probiotic (Actisaf^®^ Sc 47) through an LCA method in four trials performed during the fattening period: France (French trial), Spain (Spanish trial), and Italy (Italian trials A and B). During each trial, two groups of animals with the same characteristics were compared during the fattening period: the control group without Actisaf^®^ Sc 47 supplementation used as a baseline, and the experimental group with Actisaf^®^ Sc 47 supplementation. The calves started the trial at the ages of 224, 92, and 186 days during the French, Spanish, and Italian trials A and B, respectively. Of the sixteen impact category indicators, nine were analyzed. In Italian trials A and B, the Spanish trial, and the French trial, the use of Actisaf^®^ Sc 47 reduced CC by 4.8, 3.8, 6.6, and 5.4%; FE by 4.8, 3.9, 8.2, and 6.3%; ME by 4.9, 4.0, 6, and 6.4%; and TE by 5.3, 4.1, and 6.2%, respectively. Moreover, during the same trials, Actisaf^®^ Sc 47 reduced AC by 6.0, 4.1, 6.3, and 7.1%; LU by 4.7, 3.9, 5.8, and 6.1%; WU by 4.5, 3.4, 5.2, and 5.9%; RU (fossils) by 4.8, 3.7, 7.3, and 4.8%; and PM by 5.2, 4.1, 6.3, and 7.1%, respectively. The use of Actisaf^®^ Sc 47 has beneficial effects on the environment. Regarding Actisaf^®^ Sc 47 production, the results showed that the production of 1 kg of Actisaf^®^ Sc 47 emitted 2.1 kg of CO_2_ eq, 60% of which came from the fermentation process and 39% from drying. The results demonstrate the interest in using Actisaf^®^ Sc during the fattening period for its multiple positive outcomes: improving performance and efficiency, while reducing the global carbon footprint.

## Simple Summary

For a long time, research in animal nutrition has focused more on animal performance and health. However, due to big challenges linked to the negative effects of livestock on the environment, the scope of animal nutrition has expanded towards the search for nutritional solutions to reduce the environmental impacts of livestock production systems, especially those of ruminants. Yeast probiotics have been used for those purposes. In this study, a life cycle assessment (LCA) was used to evaluate the environmental impact of a yeast probiotic, *Saccharomyces cerevisiae* (CNCM I-4407, 10^10^ CFU/g, Actisaf^®^ Sc 47), and its use in feed rations during the fattening period of young bulls through four different trials. Supplementation with Actisaf^®^ Sc 47 at 5 g/day/animal reduced the climate change (CC) impact from 3.8 to 6.6%, particulate matter (PM) from 4.1 to 7.1%, acidification (AC) from 4.1 to 7.1%, freshwater eutrophication (FE) from 3.9 to 8.2%, marine eutrophication (ME) from 4 to 6.4%, terrestrial eutrophication (TE) from 4.1 to 7.1%, land use (LU) from 3.9 to 6.2%, water use (WU) from 3.4 to 5.9%, and fossil resource use (RU) from 3.7 to 7.3%. The beneficial effects of using Actisaf^®^ Sc 47 can be attributed to its effects on the growth performance and feed efficiency of young bulls during the fattening period.

## 1. Introduction

Among all human activities, agriculture is considered one of the main contributors to climate change due to land use, water use, and greenhouse gas emissions from livestock farming [1]. In fact, livestock production systems are responsible for 14.5% of anthropogenic greenhouse gas (GHG) emissions, which mainly result from enteric fermentation in ruminants [2]. Among ruminants, beef is at the center of concern because, apart from its effects on the environment, it contributes to the economy in different ways such as providing proteins and by-products and the creation of employment and wealth [3,4]. Beef production plays a crucial role in meeting the needs of an ever-growing population, which is estimated to reach 9.8 billion in 2030 and 11 billion in 2100, according to new United Nations projections [5]. Indeed, future demand for protein and global meat protein consumption over the next 30 years is expected to increase by 14% compared to the average consumption from 2018 to 2020 [6,7]. Improved beef production efficiency contributes to global food security and animal protein needs [8,9]. Despite its importance, environmental sustainability issues linked to beef production are acute and considered a priority by several organizations, such as the European Commission and Organization for Animal Health [8]. Compared to other animal species, beef cattle are characterized by their low efficiency in converting natural resources into edible products and have several distinct and significant impacts on the environment such as land use; water use; a high proportion of grain use, which causes soil degradation; greenhouse gas emissions; and consequently, climate change [10,11]. Beef supply chains are estimated to emit about 2.9 gigatons of CO_2_ eq, with a high contribution to global warming [12]. Such a situation puts beef cattle livestock in a paradoxical situation and forces farmers, nutritionists, scientists, and all the stakeholders to find strategies that ensure future efficient, resilient, and sustainable production systems. To deal with this situation, improving productivity and feed efficiency is an important method for continuing to provide sufficient animal protein while reducing the use of resources and the environmental impacts of beef production [13,14]. To achieve these objectives and respond to these challenges, nutrition is positioned as the most relevant and effective path. Several productivity-enhancing technologies such as growth hormones, beta-adrenergic agonists (βAAs), and ionophores have been widely used to increase animal performance but have been banned in different countries due to concerns over food safety, animal welfare, and antimicrobial resistance [15,16]. This has resulted in a growing interest in using non-conventional or “natural” feed additives, new technologies, alternatives, and novel livestock feeds that may directly or indirectly reduce environmental footprints through improvements in productivity, such as in feed efficiency, or a reduction in methane (CH_4_) production in the rumen [1,17]. Actisaf^®^ Sc 47 (Saccharomyces cerevisiae: CNCM I-4407, 10^10^ CFU/g) is produced by Phileo by Lesaffre group located Marcq-en-Barœul, Lille, France, and widely used as a feed additive to improve growth performance, digestibility, feed efficiency, and rumen metabolism [18,19,20], and, as such, it is a very good example. However, to the best of our knowledge, its effects on growth performance combined with its environmental impacts have scarcely been studied before. The effect of Actisaf^®^ Sc 47 has already been tested in dairy in our previous study using the LCA method, but this is the first test in beef cattle.

Our study was conducted to evaluate the global environmental footprint of the commercial yeast probiotic Actisaf^®^ Sc 47 from its production process to farm performance using a life cycle assessment (LCA) approach according to the ISO 14,040 [21] and 14,044 standards [22].

## 2. Materials and Methods

**Figure 1:**
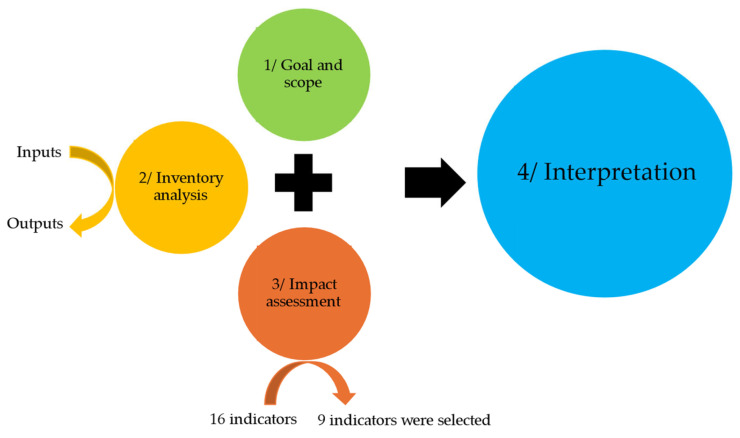
Flow diagram of the process of the LCA.

To carry out the present study based on the LCA method, we followed the ISO 14,040 [21] and ISO 14,044 [22] standards and the recommendations and requirements provided by the European Commission in the Product Environmental Footprint (PEF). The LCA includes four steps: definition of the goal and scope, life cycle inventory analysis, life cycle impact assessment, and life cycle interpretation (Figure 1).

### 2.1. Goal and Scope

The goal of the presented study was (1) to determine the environmental impacts of producing 1 kg of a yeast probiotic “Actisaf^®^ Sc 47“ produced and marketed by Phileo by Lesaffre as a feed additive in animal nutrition, and (2) to assess the environmental impacts of 1 kg of liveweight gain of young bulls with and without Actisaf^®^ Sc 47 supplementation during the fattening period. In total, nine impact categories were analyzed including CC, LU, WU, AC, ME, TE, FE, RU, and PM. The LCA was based on four zootechnical trials carried out in young bulls during the fattening period. During each trial, 2 groups of young bulls were compared: the control group without any Actisaf^®^ Sc 47 supplementation and the experimental group with Actisaf^®^ Sc 47 supplementation at the level of 5 g/cow/day. During the French trial, the Blonde d’Aquitaine breed was used at the age of 242 days. During the Spanish trial, the Holstein breed was used at the age of 92 days, and during Italian trials A and B, the Charolais breed was used at the age of 186 days. The reduction potential was calculated for each trial separately without averaging them, as the studied trials were located in different countries with different diets and living conditions.

### 2.2. System Boundary and Functional Units

**Figure 2:**
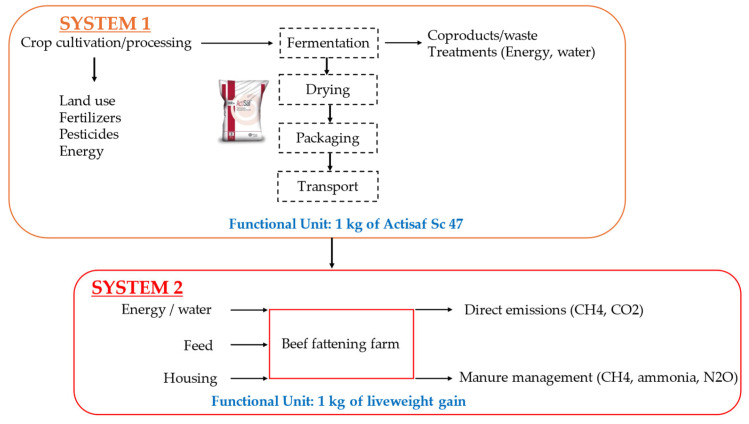
System boundary and functional units.

This study included two technical systems, the Actisaf^®^ Sc 47 production system from cradle to plant gate including all the steps from crop cultivation and processing to packaging and transportation, and the beef production system with and without Actisaf^®^ Sc 47 supplementation including all farming activities associated with animal production at the farm level (Figure 2).

The functional units chosen were 1 kg of Actisaf^®^ Sc 47 at the manufacturing level and 1 kg of liveweight gain at the farm level (Figure 2). For the system boundary of beef production, energy use at the farm, imported feed, feed produced at the farm, and emissions from soil application were considered.

### 2.3. Inventory Analysis and Input Data

Data for the LCA of Actisaf^®^ Sc 47 production including feed cultivation, processing, quantity of energy, quantity of water, and type of packaging were provided by the Lesaffre factory based in the north of France. Background data related to transportation and the production and delivery of energy were obtained from AGRIBALYSE v3.0.1 (2020) and ecoinvent v3.6 (2020). All the data linked to the different steps followed to produce Actisaf^®^ Sc 47 from the reception at the factory to the storage were provided by the company based on data from 2021. At the Lesaffre plant, the Actisaf^®^ Sc 47 production process starts with a fermentation step in which a specific yeast strain is inoculated into a nutrient-rich fermentation medium containing a sugar source, such as molasses, as well as sources of nitrogen, minerals, and other nutrients essential for its growth. Yeast fermentation takes place in fermenters where the temperature, pH, oxygen levels, and input of ingredients are controlled. The outputs of this step are the yeast cream, as well as by-products that are revalorized and water that is treated on site. The yeast cream produced is then centrifuged, filtered, and dried to produce yeast powder.

Primary data including the feed composition and zootechnical performance used to estimate the environmental impacts of producing 1 kg of liveweight gain with and without Actisaf^®^ Sc 47 supplementation came from Phileo. The effects of Actisaf^®^ Sc 47 on growth performance, intake, and feed efficiency were obtained from 4 trials conducted on 4 different farms. The trials were conducted in countries with a high production of beef across Europe and which are characterized by different production systems including breed, farming (extensive vs. intensive), and feeding practices. During each trial, 2 groups of fattening bulls were compared: the control group without any Actisaf^®^ Sc 47 supplementation and the experimental group with Actisaf^®^ Sc 47 supplementation at a recommended dose of 5 g/d/calf (Table 1 and Table 2). During all the trials, Actisaf^®^ Sc 47 was diluted in a premix and then mixed with a complete feed. Secondary or generic data related to manure and energy were obtained from AGRIBALYS. Feed ingredients either imported or produced at the farm were considered for the calculation.

**Figure 3:**
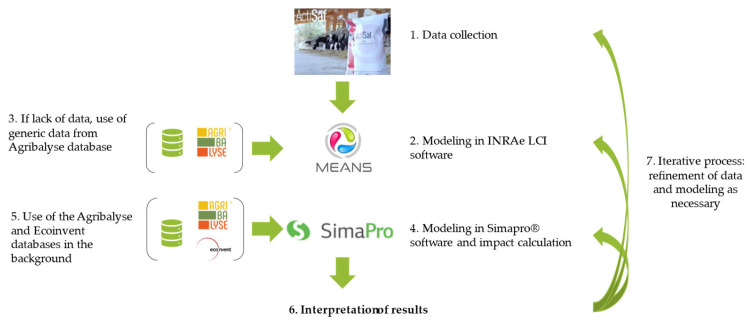
Steps of modeling.

For other zootechnical data, AGRIBALYSE, a reference database of environmental impact indicators for agricultural products produced in France, was used as the reference for comparison and adapted to represent performances obtained with the use of the additive using the MEANS InOut software V4.2, which implements recommended methodologies defined for the AGRIBALYSE program (Figure 3). Other data related to animal characteristics such as growth physiology, early breed, late breed, energy input, water input, and bedding material were obtained from the French national platform “MEANS” (MulticritEria AssessmeNt of Sustainability). The quantity of manure was calculated using the composim tool used to calculate the quantity and composition of livestock effluents (https://ifip.asso.fr/documentations/4302-composim-calculateur-de-la-quantite-et-de-la-composition-des-effluents-delevage/ accessed on 10 January 2013).

### 2.4. Life Cycle Impact Assessment

The modeling of the environmental impacts of Actisaf^®^ Sc 47 production and the four animal trials for both control and experimental groups was performed using SimaPro 9.3. Over the 16 indicators defined by the European Commission, 9 indicators were analyzed during our study: climate change, land use, particulate matter, water use, terrestrial and freshwater acidification, eutrophication, and fossil resource use.

## 3. Results

### 3.1. Environmental Impacts of Actisaf^®^ Sc 47 Production

**Figure 4:**
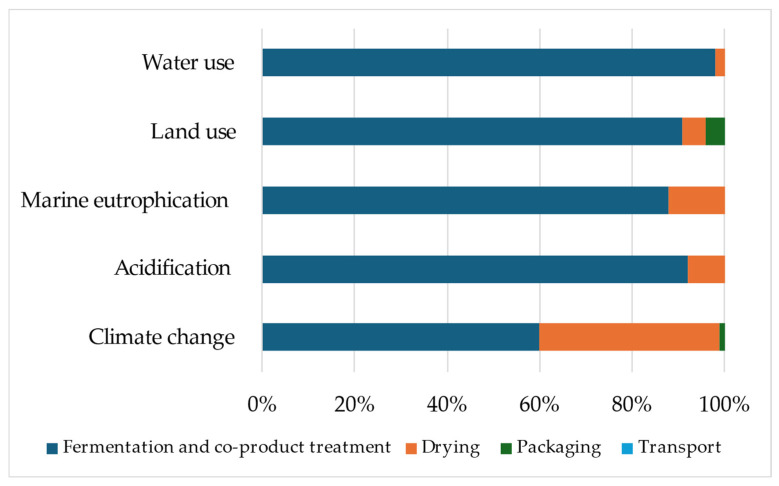
Contribution (%) of the subsystems to the potential environmental impacts of the production of 1 kg of Actisaf^®^ Sc 47.

The environmental impacts generated by the production of 1 kg of Actisaf^®^ Sc 47 at the Lesaffre factory were published in our last paper [23]. The production of 1 kg of Actisaf^®^ Sc 47 generated 2.10 kg of CO_2_ eq (Figure 4), in which 60, 37, 1, and 2% were emitted during the fermentation process, drying, packaging, and transport, respectively. The same tendencies were observed for land use, water consumption, acidification, and eutrophication with a high contribution of the fermentation process followed by drying (Figure 4).

### 3.2. Environmental Impacts of 1 kg of Liveweight Gain With and Without Actisaf^®^ Sc 47

**Figure 5:**
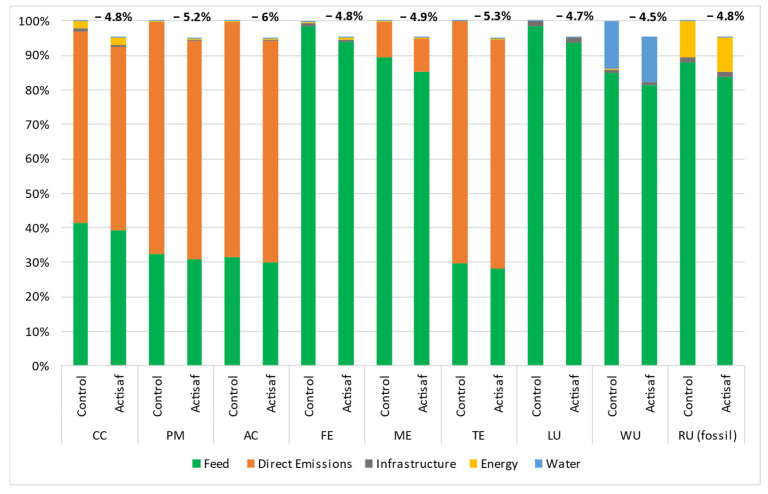
Environmental gain due to the use of Actisaf^®^ Sc 47 in fattening beef production during Italian trial A. CC: climate change; PM: particulate matter; AC: acidification; FE: freshwater eutrophication; ME: marine eutrophication; TE: terrestrial eutrophication; LU: land use; WU: water use; RU: resource use (fossil).

**Figure 6:**
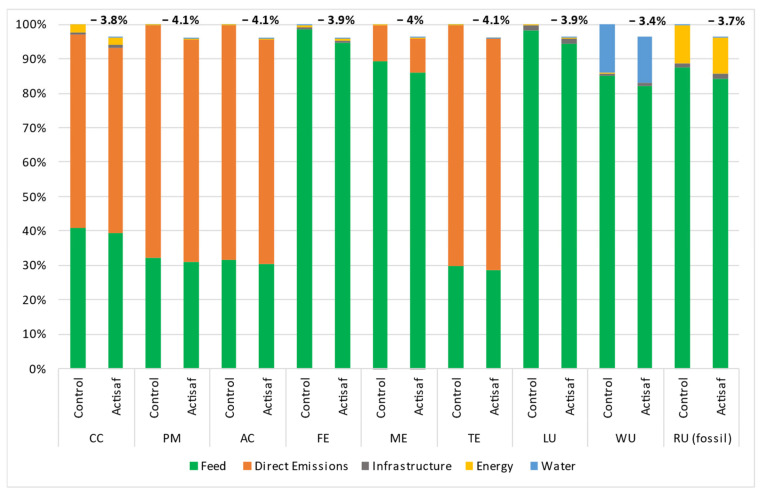
Environmental gain due to the use of Actisaf^®^ Sc 47 in fattening beef production during Italian trial B. CC: climate change; PM: particulate matter; AC: acidification; FE: freshwater eutrophication; ME: marine eutrophication; TE: terrestrial eutrophication; LU: land use; WU: water use; RU: resource use (fossil).

**Figure 7:**
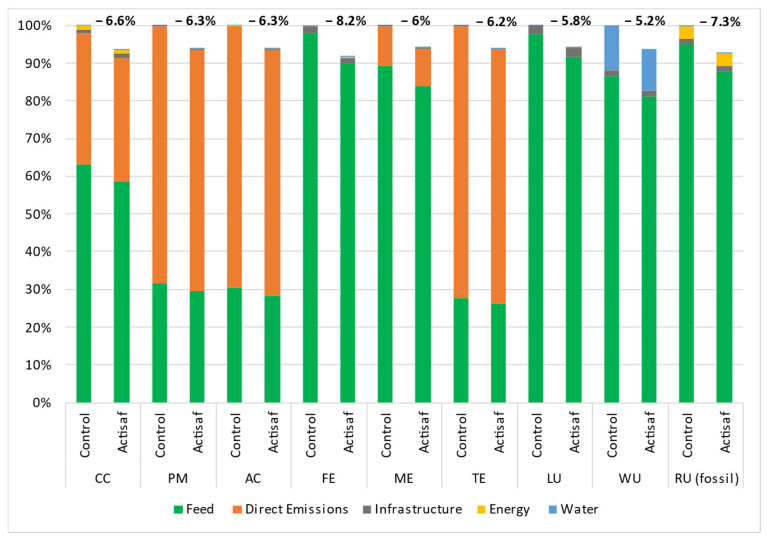
Environmental gain due to the use of Actisaf^®^ Sc 47 in fattening beef production during the Spanish trial C. CC: climate change; PM: particulate matter; AC: acidification; FE: freshwater eutrophication; ME: marine eutrophication; TE: terrestrial eutrophication; LU: land use; WU: water use; RU: resource use (fossil).

**Figure 8:**
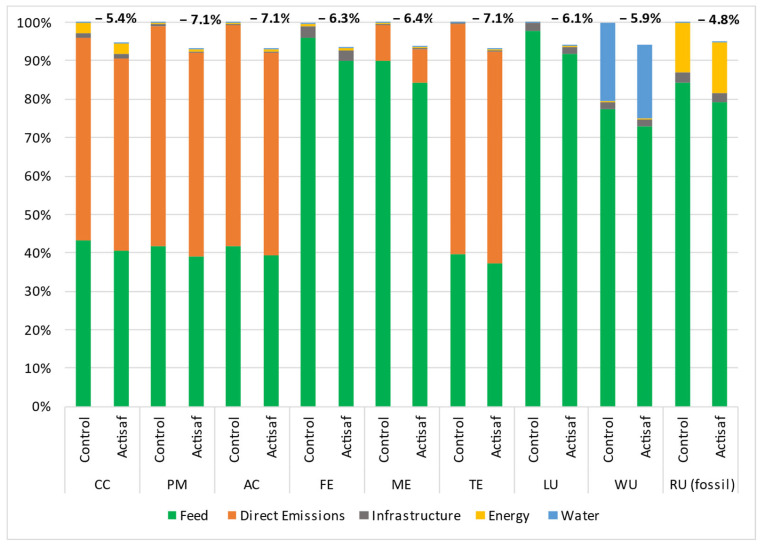
Environmental gain due to the use of Actisaf^®^ Sc 47 in fattening beef production during the French trial D. CC: climate change; PM: particulate matter; AC: acidification; FE: freshwater eutrophication; ME: marine eutrophication; TE: terrestrial eutrophication; LU: land use; WU: water use; RU: resource use (fossil).

The data used came from four trials carried out in different countries with different management systems such as the rations, animal breed, duration, and climatic conditions. Thus, the environmental impacts were calculated for each trial separately to give a range of variation and a global mean value for each impact category. The environmental benefits that resulted from the use of Actisaf^®^ Sc 47 in fattening bulls during Italian trial A (trial A), Italian trial B (trial B), the Spanish trial C (trial C), and the French trial (trial D) compared to standard conditions are shown in Figure 5, Figure 6, Figure 7 and Figure 8, respectively. In general, the effect of Actisaf^®^ Sc 47 on the environmental impacts was very similar between all the trials. The supplementation with Actisaf^®^ Sc 47 showed a positive impact on all the environmental categories. All four assessed trials showed a reduction in CC impact with a range from 3.8 to 6.6% (mean reduction = 5.15%), PM from 4.1 to 7.1% (mean reduction = 5.67%), AC from 4.1 to 7.1% (mean reduction = 5.87%), FE from 3.9 to 8.2% (mean reduction = 5.80%), ME from 4 to 6.4% (mean reduction = 5.32%), TE from 4.1 to 7.1% (mean reduction = 5.68%), LU from 3.9 to 6.1% (mean reduction = 5.12%), WU from 3.4 to 5.9% (mean reduction = 4.75%), and RU (fossil) from 3.7 to 7.3% (mean reduction = 5.15%). For all the trials, direct emissions were the main contributors to CC, PM, AC, and TE followed by feed ingredients, which were the main contributors to FE, ME, LU, WU, and RU, respectively.

**Figure 9:**
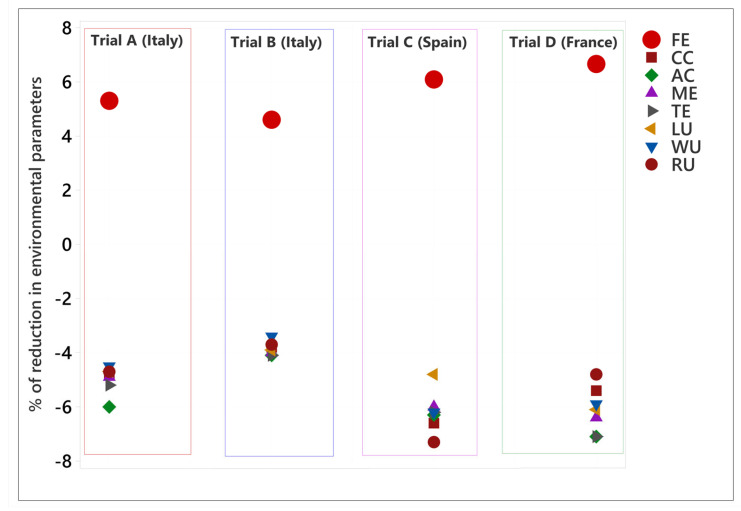
Relationship between impact evolution and FE variation. FF: feed efficiency; CC: climate change; AC: acidification; ME: marine eutrophication; TE: terrestrial eutrophication; LU: land use; WU: water use; RU: resource use (fossil).

**Figure 10:**
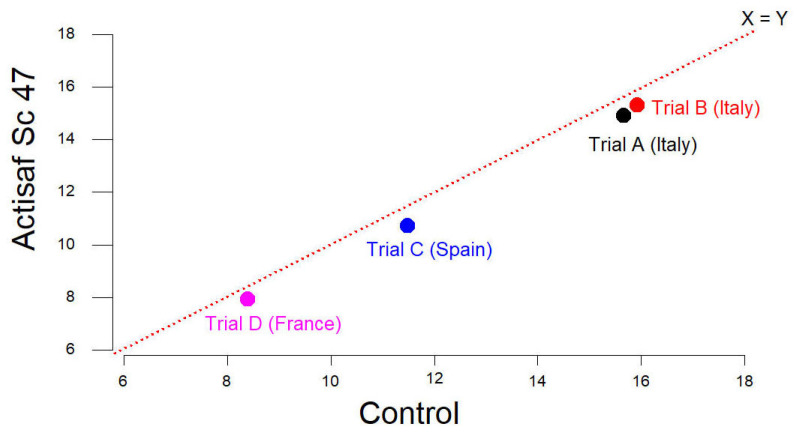
Effect of Actisaf^®^ Sc 47 on CC compared to the control (kg CO_2_ eq/kg LW gain).

The environmental gains in the different trials resulted mainly from the better feed efficiency. The animals that received Actisaf^®^ Sc 47 consumed less feed to produce 1 kg of liveweight gain compared to the control. A positive correlation between feed efficiency (FE) and the % reduction in environmental impacts was observed, as shown in Figure 9. The % increase in FE was 5.3, 4.60, 6.09, and 6.66% in trials A (Italy), B (Italy), C (Spain), and D (France), respectively. The comparison between trials indicated that when FE increases, CC decreases, as indicated in Figure 10.

The higher % increase in FE calculated according to Equation 1 allowed by Actisaf^®^ Sc 47 in the French trial (D), followed by the Spanish trial (C), Italian trial (A), and Italian trial (B), can explain the lower CC during the corresponding trials, as indicated in Figure 10.

(1) $$\%\;of\;increase\;in\;FE\;=\;\left(\frac{FE\;with\;Actisaf\;-\;FE\;with\;control}{FE\;with\;control}\right)\;\times \;100$$

## 4. Discussion

Identifying solutions that increase animal performance and reduce environmental impacts has become a major concern across the world and constitutes an important challenge for the future. This challenge is particularly pronounced for beef production because it requires a lot of land, water, and energy and generates considerable waste and greenhouse gases such as CH_4_, ammonia, and nitrous oxide (N_2_O), which contributes to climate change and warming [24,25]. It is thus essential to improve its sustainability globally. Evaluating the environmental impacts is a good approach to understand and define the best strategies that allow a more sustainable production system to be built with low environmental impacts for current and future generations. Thus, LCA is widely used to calculate the impact of any type of product including all stages of the chain from production to use. The aim of this study was to quantify the environmental impacts of a yeast probiotic produced by Phileo by Lesaffre and used as a feed additive in animal nutrition. Indeed, four different trials were used to estimate the potential effects of producing 1 kg of liveweight gain with and without using Actisaf^®^ Sc 47.

Regarding the production process, the environmental impacts of producing Actisaf^®^ Sc 47 were recently published in our LCA study conducted in dairy cows [23]. The production of 1 kg of Actisaf^®^ Sc 47 emitted 2.10 kg of CO_2_ eq in which 1.26 kg of CO_2_ eq was emitted during fermentation, which may be explained by the high substrate demand of this step.

Regarding the use of Actisaf^®^ Sc 47 in beef cattle during the fattening period which corresponds to the objectives of the four trials used in our study, feed and direct emissions were identified as the main factors responsible for environmental impacts for all the analyzed categories. Our results agree with those of [26,27], who found that feed production contributes the most to the greenhouse gas emissions and consequently the carbon footprint. By comparing different diets, [26] observed that feed and enteric CH_4_ emissions accounted for 47.9 and 26.7% of the total carbon footprint.

The objectives of using live yeast were mainly oriented towards increasing zootechnical performance such as growth, digestibility, and feed efficiency, and no study has been published on environmental impacts particularly in beef cattle. Our results showed that using 5 g of Actisaf^®^ Sc 47 in beef cattle during the fattening period provided benefits to the nine environmental categories compared to standard farming. Regarding CC, the main contributor was direct emissions from enteric fermentation for the two Italian and the French trials. Similar observations were reported by [28], who estimated that enteric fermentation contributed 47% to the fattening phase. However, the main contributor for the Spanish trial was feed. The trials used during this study were conducted in countries with a high production of beef across Europe and which are characterized by different production systems including breed, farming (extensive vs. intensive), and feeding practices. The difference between the trials can be explained by the difference in digestibility which depends on the type of diet. The diet used during the Spanish trial contained a lower percentage of forages and straw, which promoted better digestibility and lower emissions compared to the diets used during the three other trials. Indeed, Ref. [29] observed high enteric fermentation emissions with a roughage diet in the grazing system compared to a concentrate-based diet in the landless system with equivalent nutritional value. Supplementation with Actisaf^®^ Sc 47 reduced CC, which is mainly affected by diet and direct emissions from enteric fermentation [26]. The impact of the contribution of both categories was reduced by Actisaf^®^ Sc 47. The beneficial effect of Actisaf^®^ Sc 47 on CC can be explained indirectly by its positive effect on growth performance and feed efficiency. Indeed, compared to the control, animals that received Actisaf^®^ Sc 47 consumed less feed to produce the same liveweight gain. The use of Actisaf^®^ Sc 47 in ruminants is well documented and several studies have shown its positive effects on rumen metabolism, growth performance, digestibility, and feed efficiency [30,31,32,33]. The direct effect of Actisaf^®^ Sc 47 on methane emissions has not been studied before, but we hypothesized that it may slightly reduce CH4 through the stimulation of reductive acetogens that serve as hydrogen sinks by directing it towards the production of acetate instead of methane [34]. Using the LCA approach to evaluate the impacts of feed additive on swine, broiler chicken, and turbot, Refs. [35,36] identified an improvement in feed efficiency as key to reducing climate change.

Compared to the control, the production of 1 kg of liveweight of animals supplemented with Actisaf^®^ Sc 47 generated less terrestrial and water acidification and PM, which are linked to the emissions of ammonia and N_2_O from manure management [37,38], followed by feeding as indicated by [39]. Both ammonia and N_2_O excretion depend on the rumen environment and metabolism, as a large part of the ingested nitrogen is lost in urine and feces due to the low nitrogen-use efficiency of beef cattle [40]. Thus, it seems important to increase the nitrogen-use efficiency to reduce ammonia and N_2_O and consequently AC and PM [41]. The mechanism by which Actisaf^®^ Sc 47 reduced AC and PM may be attributed to its positive effect on nitrogen-use efficiency. By its effect on ruminal metabolism, particularly the stimulation of fibrolytic bacteria [42,43,44] that have a strong preference for ammonia [45], Actisaf^®^ Sc 47 allows an increase in ammonia utilization for microbial protein synthesis, as well as overall nitrogen utilization, and consequently induces a reduction in urinary nitrogen excretion and ammonia emission [46]. The authors of [47] observed a positive correlation between the ammonia concentration in the rumen and the ammonia concentration in manure. The low AC allowed by Actisaf^®^ Sc 47 can also be explained by its effect on feed production, which necessitates the use of fertilizers. The authors of [48] compared four beef production systems and concluded that the ammonia emissions from fertilizer application to the feed crop are a source of AC. The reduction in AC caused by Actisaf^®^ Sc 47 can be explained by its reducing effect on the carbon footprint, which is directly linked to greenhouse gas emissions such as carbon dioxide (CO_2_), CH_4_, and N_2_O. A positive correlation was observed between greenhouse gas emissions and the acidification of soil and water [49,50].

Feed production accounted for most of the freshwater and marine eutrophication during all the trials as observed by [38,39,40,41,42,43,44,45,46,47,48]. Fertilizers and herbicides are the major pollutants from feed production and contribute greatly to water and marine eutrophication. This impact is reduced by using Actisaf^®^ Sc 47 probably due to the use of less feed to produce 1 kg of liveweight gain. Eutrophication can result from an excess of organic compounds such as nitrogen and phosphorus in manure, as explained by [51]. The direct effect of Actisaf^®^ Sc 47 on reducing eutrophication has not been previously studied but may be attributed to the improvement in nitrogen and phosphorus utilization and the reduction in phosphorus excretion in manure via the stimulation of growth of phytase-producing bacteria such as *Selenomonas ruminantium*, *Megasphaera elsdenii,* and *Prevotella* sp. [52,53].

The use of Actisaf^®^ Sc 47 resulted in a lower impact potential for LU. Feeding inputs were the main contributor of LU during all the trials and were reduced by using Actisaf^®^ Sc 47. The beneficial effect of Actisaf^®^ Sc 47 on LU can be explained by the high growth performance and better feed efficiency of calves compared to the control. Our results agree with those observed by [38] who compared the concentrate-based farming system and the grazing system and attributed the low land use to the concentrate-based system due to the better growth performance and feed efficiency. The same conclusions on the negative correlation between feed efficiency and CC, LU, WU, AC, and eutrophication were mentioned for beef [54], dairy [55,56,57], broilers, and swine [35]. During the four trials analyzed in our study, Actisaf^®^ Sc 47 led to an increase in feed efficiency, which explains its global beneficial effects.

Water is the source of life, and water use must be controlled, particularly in agriculture, which necessitates a significant quantity. In livestock, water is used for drinking, irrigation, cooling, and cleaning. In beef, the global average water footprint is higher than those of other animal species and ranges from 15,415 to 14,597 L/kg of beef [58,59]. Increasing water use efficiency, saving water, and reducing the water footprint are part of sustainability. The main contributors of water use during the four trials were feed production and water consumption and both were reduced by using Actisaf^®^ Sc 47. The mechanism by which Actisaf^®^ Sc 47 reduced water use is unknown as no study has evaluated this effect, but it can be explained in two ways. The reduction in water use by Actisaf^®^ Sc 47 can be attributed to its positive effect on feed efficiency, as indicated by the authors of [60], who identified a positive association between feed efficiency and water intake in beef. As observed in Figure 5, Figure 6, Figure 7 and Figure 8, the use of Actisaf^®^ Sc 47 reduced the use of land to produce food and therefore the quantity of water needed for irrigation, which translates into a reduction in water use.

Our current results agree with our latest results obtained in dairy cows. Indeed, using an LCA approach, we observed a reduction in the carbon footprint, acidification, eutrophication, and land and water use with Actisaf^®^ Sc 47 [23].

The life cycle assessment approach was used to evaluate the environmental impacts of different feed additives used in animal nutrition and showed about a 10% improvement in the environmental footprint due first to their positive effects on animal performance, feed efficiency, and greenhouse gas emissions [61]. The use of Actisaf^®^ Sc 47 reduced CC from 3.8 to 6.6% during the fattening period. The difference from the value mentioned by [56] can be explained by the proper mode of action of each additive, the dose, the direct effect on environmental parameters such as CH_4_ and ammonia, or indirect effects such as improving animal health, fertility, and efficiency. An LCA study carried out on dairy cows supplemented with Actisaf^®^ Sc 47 showed a beneficial effect on the carbon footprint with a reduction of 5% during the supplementation period. As mentioned previously, no LCA was carried out to evaluate the environmental impact of live yeast in beef cattle. The use of Actisaf^®^ Sc 47 as a fermentation-based solution might be of high interest for different reasons: better growth performance, better feed efficiency, lower environmental impacts, and, consequently, better economic gains for the farmers. Our study only included the fattening period, which may constitute a slight limitation, and an analysis of the entire production cycle seems to be an interesting approach.

## 5. Conclusions

Actisaf^®^ Sc 47 has been used as a feed additive in animal nutrition for several years, especially for its positive effects on rumen metabolism and zootechnical performance such as digestibility, growth, and feed efficiency. However, its direct effects on the environmental impacts of beef cattle have not been analyzed before. An LCA study on Actisaf^®^ Sc 47 production and its use in beef cattle during the fattening period was herein performed to assess its environmental impact by analyzing different categories. The production of 1 kg of Actisaf^®^ Sc 47 emitted 2.1 kg of CO_2_ eq. On average, in fattening beef cattle, the use of Actisaf^®^ Sc 47 during the four trials reduced climate change, particulate matter, acidification, freshwater eutrophication, marine eutrophication, terrestrial eutrophication, land use, water use, and resource use (fossil) by 5.15, 5.67, 5.87, 5.80, 5.32, 5.68, 5.12, 4.75, and 5.15%, respectively. Similar results were obtained in dairy cows using the LCA approach with a 5% reduction in climate change brought about by Actisaf^®^ Sc 47. Hence, due to multiple positive effects, the use of Actisaf^®^ Sc 47 is highly recommended in beef cattle during the fattening period. Other benefits, such as improvements in health, reductions in morbidity and mortality, and manure quality for each group, were not included in this study, suggesting a higher impact reduction.

## Figures and Tables

**Table 1:** Zootechnical performance during all the trials.

	Trial A		Trial B		Trial C		Trial B	
Place	Italy		Italy		Spain		France	
Year	2020		2019		2020		2002	
Breed	Charolais		Charolais		Holstein		Blonde d’Aquitaine	
Group	CON	Actisaf	CON	Actisaf	CON	Actisaf	CON	Actisaf
Number	70	70	70	70	55	55	87	87
Duration (day)	186	186	186	186	92	92	226.2	222.2
IBW (kg)	400	401	415.6	415.6	349	348	320	323.9
FBW (kg)	681	697	681.54	692.48	488	491	660	673.7
BWG (kg)	281	296	266	277	139	143	340	350
ADG (kg/d)	1.51	1.59	1.43	1.49	1.52	1.57	1.50	1.47
DMI (kg/d/calf)	11.5	11.5	10.98	10.98	7.69	7.45	7.93	7.79
FCR	7.61	7.22	7.67	7.37	5.05	4.75	5.27	4.94
MQ (ton/year)	217	217	227.5	227.5	50.6	52.08	163.35	161
CY (%)	59.8	59.7	59.3	59.4	52.5	52.7	64.8	64.2
Meat price (EUR/kg carcass)	5.8	5.8	5.8	5.8	4.8	4.8	5.2	5.2

CON: control group; IBW: initial body weight; FBW: final body weight; DMI: dry matter intake; BWG: body weight gain; FCR: feed conversion ratio (DMI/kg average daily gain); MQ: manure quantity; CY: carcass yield.

**Table 2:** Feed composition during all the trials.

	Trial A (Italy)		Trial B		Trial C		Trial B	
Place	Italy		Italy		Spain		France	
Breed	Charolais		Charolais		Holstein		Blonde d’Aquitaine	
Ration (%)	CON	Actisaf	CON	Actisaf	CON	Actisaf	CON	Actisaf
Corn silage	48.6	48.6	49	49				
Wheat straw	6.3	6.3	6	6			11.4	11.4
Corn meal	24.3	24.3	24	24	49.98	49.98	68.2	68.2
Wheat bran	6.3	6.3	6	6				
Grape marc	4.6	4.6	5	5				
Sunflower meal	3.1	3.1	3	3				
Rape cake	2.5	2.5	3	3				
Soybean meal	2.5	2.5	3	3	6.22	6.22	17	17
Coproduct of glutamic acid	0.3	0.3	0	0				
Urea	0.3	0.3	0	0	0.5	0.5		
Mineral–vitamin	1.2	1.2	1	1	2.3	2.3	3.4	3.4
Wheat middling					8	8		
Soybean hulls					9.2	9.2		
Barley grain meal					12	12		
Corn DDG					4	4		
Palm oil					2.8	2.8		
Beet pulp					5	5		

CON: control group.

## Data Availability

All data stemming from the present research are enclosed in the tables and figures. Any additional data will be made accessible from the corresponding authors upon reasonable request.
